# Nutrient recovery from treated municipal wastewater for basil production in indoor farming

**DOI:** 10.3389/fpls.2026.1919603

**Published:** 2026-09-17

**Authors:** Reyhaneh Gorji, Xiaolin Wang, Yahya Jani, Sebastian Schwede, Monica Odlare

**Affiliations:** Future Energy Center, Department of Engineering Sciences, Mälardalen University, Västerås, Sweden

**Keywords:** basil production, controlled environment agriculture, indoor farming, nutrient recycling, wastewater reuse

## Abstract

Climate change and population growth are driving the increasing demand for alternative water sources in agriculture. Nutrient-rich treated municipal wastewater emerges as a potential alternative for agriculture production. Therefore, a pot experiment using zeolite as the growing substrate to simulate hydroponic conditions was conducted to assess the impacts of fully treated municipal wastewater and partially treated wastewater that undergone only mechanical treatment step on basil (*Ocimum basilicum* cv. Edwina) production, aiming to explore the potential of nutrient recovery from these wastewater sources. Tap water and optimal liquid fertilizer were utilized as negative and positive controls, respectively. Results showed that primarily treated wastewater significantly enhanced basil production compared to the tap water, although the large relative increase was partly attributable to the extremely low biomass under tap-water irrigation. However, with consistently greater production and nutrient concentrations produced by the positive control, nutrient supplementation was still required in the wastewater treatments to achieve greater production and nutrient levels comparable to those with complete mineral nutrient solution. Analysis of selected heavy metals indicated no evidence of excessive accumulation in basil shoots under the conditions of this study, although comprehensive food safety assessment would require evaluation of additional contaminants, including microbiological hazards and emerging pollutants. The findings provide insights into the potential of treated municipal wastewater reuse for nutrient recovery in controlled agricultural systems.

## Highlights

- Primary treated wastewater can partially supply nutrients for basil production
- Metal uptake and translocation are dependent on wastewater composition
- Selected metal concentrations resulted in low non-carcinogenic risks under tested conditions
- Wastewater reuse offers a pathway toward circular nutrient management

## 1 Introduction

Water scarcity is a critical global challenge driven by population growth, urbanization, and economic development (Al-Karaki, 2011; Magwaza et al., 2020). The increasing demand for freshwater has led to a surge in wastewater generation, highlighting the need for sustainable water management (Karef et al., 2025; Qadir et al., 2020). Treated wastewater reuse addresses multiple simultaneously benefits including enhancing water availability, recycling essential nutrients, and reducing the environmental footprint associated with synthetic fertilizer production and wastewater treatment (Hofmann et al., 2024; Qadir et al., 2020). Treated wastewater often contains macronutrients such as nitrogen (N), phosphorus (P), and potassium (K), as well as beneficial micronutrients, making it a potential alternative nutrient source that may partly reduce dependence on conventional fertilizer (Cifuentes-Torres et al., 2021; Karef et al., 2025).

Controlled environment agriculture (CEA) systems such as hydroponics offer a unique opportunity to test wastewater reuse under standardized conditions (Cifuentes-Torres et al., 2021; Hofmann et al., 2024). Hydroponics allows precise control over growing conditions, eliminating soil variability and enabling focused assessment of nutrient uptake, plant health, and contaminant transfer (Ma et al., 1997). Moreover, hydroponic systems can purify and remediate wastewater through plant nutrient uptake, making them an effective solution for both nutrient supply and wastewater treatment (Cifuentes-Torres et al., 2021). While hydroponics has been widely used to study plant responses to nutrient availability and stress factors, soilless pot experiments using inert media such as zeolite can effectively simulate hydroponic conditions while allowing easier handling of solid-phase residues and roots.

Prior studies have demonstrated the potential of treated wastewater in soilless systems for supporting plant growth. For example, Al-Karaki (2011) showed that tertiary treated sewage wastewater increased barley fodder yields significantly compared to tap water while maintaining safe levels of heavy metals. Similarly, Adrover et al. (2013) found that while treated wastewater supplies essential nutrients, additional fertilization may be required to maintain high productivity. However, much of the existing literature has focused on secondary- or tertiary-treated effluents, the remediation of wastewater by plants, or crop growth alone. Comparatively less attention has been given the trade-off created by wastewater treatment itself: whether retaining nutrients in an earlier treatment-stage stream can improve crop nutrient recovery compared with final treated effluent, and how this difference affect crop productivity, plant nutrient allocation, metal uptake, and substrate retention within the same controlled cultivation system.

One major concern related to reclaimed wastewater for irrigation is the potential health risks associated with the uptake of metals and other elements by plants (Karef et al., 2025; Qadir et al., 2010). The accumulation of toxic metals and micropollutants in the edible parts of plants can possess health challenges if the concentrations of these elements exceed guidelines limits (Ali and Ahirwar, 2025; Narwal et al., 2023). The extent to which crops absorb, transfer and accumulate metals differs due to the differences in physiological metabolism (Ali and Ahirwar, 2025; Säumel et al., 2012; Zhang et al., 2018). Therefore, a health risk assessment analysis can be a way for identifying potential risk when using reclaimed wastewater for irrigation.

The present study therefore evaluates two wastewater streams that represent contrasting stages of municipal treatment: (i) a primarily treated wastewater (PW) stream collected after mechanical (primary) treatment but prior to biological processing, and (ii) a reclaimed final effluent (RW) stream representing the fully treated, discharge-ready effluent. PW was selected instead of a secondary-treated stream because biological treatment is designed to remove organic matter and, depending on the treatment configuration, substantial fractions of nitrogen and phosphorus. Sampling before biological treatment therefore allowed a stream expected to retain a larger proportion of potentially recoverable nutrients to be investigated. RW was included as a conservative and extensively treated contrast: it represents a low-nutrient, lower-risk endpoint of treatment that could still provide residual nutrients and useful background information for assessing the benefit–risk tradeoffs of reuse in controlled-environment agriculture. Selecting PW and RW also lets the experiment address two practical pathways for circular nutrient use in urban food systems. PW is attractive where the aim is direct nutrient recycling with modest on-farm processing to meet crop demand. RW models the scenario in which reclaimed water is used primarily for water substitution to drinking and tap water. Comparing the two streams permits evaluation of (a) growth performance and nutrient uptake under differing nutrient loads, and (b) associated food-safety implications (metal uptake and translocation) that are likely to differ by stream quality.

Within the European Union, agricultural reuse of treated urban wastewater is governed by Regulation (EU) 2020/741 (European Parliament and Council of the European Union, 2020), which establishes minimum requirements for reclaimed-water quality, monitoring, permitting, and risk management. Because PW had undergone only primary treatment, it did not meet the treatment requirements for commercial irrigation of raw-consumed crops and was therefore evaluated only as an experimental nutrient-rich stream under controlled conditions. From a practical perspective, examining PW makes it possible to assess whether nutrients could, in principle, be recovered closer to their point of entry into the treatment process, before energy- and resource-intensive nutrient removal has occurred. However, PW is not proposed here as irrigation water suitable for direct commercial food production. Any practical application would require additional treatment, validated pathogen reduction, nutrient balancing, monitoring of relevant chemical contaminants, and verification of compliance with applicable water-reuse and food-safety requirements (European Parliament and Council of the European Union, 2020).

Finally, because the reuse of municipal streams must account for both agronomic and public-health constraints, this study couples growth and nutrient analyses with assessments of bioconcentration factor (BF), translocation factor (TF) and target hazard quotient (THQ) for key metals (Hussain et al., 2022; Liu et al., 2023; Nag and Cummins, 2022). This combined approach allows us to evaluate whether nutrient recovery from municipal streams is agronomically viable and whether it poses measurable non-carcinogenic health risks under the test conditions used in this study.

The novelty of the present study lies in the integrated comparison of primary-treated wastewater, final treated effluent, tap water, and a conventional nutrient solution within the same zeolite-based pot system simulating hydroponic cultivation under standardized indoor conditions. This design enables streams with markedly different nutrient compositions to be evaluated against both a nutrient-deficient control and a nutritionally optimized control. By combining plant-growth measurements with analyses of macro- and micronutrients in irrigation water, shoots, roots, and the post-cultivation substrate, as well as metal-specific BF, TF, and THQ, the study provides knowledge about nutrient and selected-metal distribution across the water-substrate-plant system. The study therefore extends previous work by examining not only whether treated wastewater can support plant growth, but also how the treatment stage influences nutrient recovery, plant uptake, and the limitations that must be addressed before such streams can be considered for controlled-environment crop production.

## 2 Materials and methods

### 2.1 Experimental setup

In this experiment, Genovese basil (*Ocimum basilicum* cv. Edwina) was cultivated in round pots with dimensions of 15 cm in diameter and 13.9 cm in height. Each pot was filled with 1.2 L of zeolite substrate (Lechuza Basic Pon), simulating hydroponic conditions. Initially, twelve seeds (Olssons Frö AB, Sweden) were sown in each pot. After germination, ten healthy and uniform seedlings were retained. Each treatment included three independent pots as biological replicates (n = 3). At harvest, plant material from the ten plants within each pot was pooled to form one composite sample. Each composite sample was analyzed separately for nutrient and metal concentrations; therefore, the pot was considered the experimental unit for statistical analysis The experimental setup was conducted under controlled indoor conditions, with temperature maintained between 18-20 °C and humidity levels at 40-50%. LED lights were utilized to provide illumination, delivering a photosynthetic photon flux density (PPFD) of 220 μmol·s^-^¹·m^-^² (18-h light/6-h dark) in the light spectrum range of 350–800 nm. Pots positions were randomly changed after each irrigation during the experiment to ensure that positional effects inside the growth chamber did not influence plant performance. The experiment spanned from 24 April to 28 June, 2023. Different water sources or nutrient solutions were applied in 100 mL every two days.

### 2.2 Irrigation water and fertilizer characterization

Four distinct water streams were incorporated for irrigation: tap water (utilized as the negative control), optimal liquid fertilizer (serving as the positive control), reclaimed effluent water, and primarily treated wastewater, abbreviated as TAP, NUT, RW, and PW respectively. The RW and PW waters were sourced from a municipal wastewater treatment plant (WWTP) Mälarenergi AB, Västerås. The inclusion of PW and RW aimed to explore the performance of different qualities of wastewater in hydroponic systems. PW, collected after mechanical treatment but prior to biological treatment, was selected for its relatively high nutrient content. This water stream represents a potential source of nutrients that has undergone initial treatment steps, reducing its risk compared to raw sewage or reject stream, while still retaining significant nutrient value. RW, on the other hand, represents fully treated effluent water, with substantially lower nutrient concentrations. It was chosen not only as a contrast to PW, but also to TAP, allowing for the assessment of whether minimal residual nutrients in reclaimed water can support plant growth compared to a nutrient-free baseline. The optimal liquid fertilizer solution was provided by the vertical farming company (Swegreen AB, Sweden). This solution was formulated by dissolving a blend of various chemical compounds in water, supplying both macro- and micronutrients essential for plant growth. The resulting solution had an electrical conductivity (EC) of 1.4 mS/cm, indicating its balanced concentration of dissolved nutrients for hydroponic cultivation. Water sample analysis was conducted externally at Eurofins Scientific SE, including the determination of key parameters such as the concentrations of Phosphorus (P), Nitrogen (N), Potassium (K), and various metals, including Arsenic (As), Barium (Ba), Lead (Pb), Cadmium (Cd), Cobalt (Co), Copper (Cu), Chromium (Cr), Mercury (Hg), Nickel (Ni), Vanadium (V), and Zinc (Zn). The irrigation volume was maintained constant throughout the experiment, with 100 mL of the respective irrigation water applied to each pot every other day, regardless of plant growth stage. All treatments therefore received the same total irrigation volume. Each pot was placed on an individual collection tray. Any leachate draining from the pot was retained in its corresponding tray and was neither collected nor discarded. The leachate was no longer visible by the following day, suggesting that it had been reabsorbed into the substrate and root zone. The composition of irrigation water is shown in **Table 1**. The pH was analyzed using a pH meter Metrohm model 744.

**Table 1:** Composition of different irrigation sources used in this experiment.

Parameters/Unit	Irrigation water			
	TAP	NUT	RW	PW
pH	7.7	6	7.1	7.3
P (mg L^-1^)	0.01015	65.5	0.185	2.5
N (mg L^-1^)	0.61	220	15.0	31
K (mg L^-1^)	2.3	225	13.5	17
As (mg L^-1^)	0.000285	0.0007	0.0003	0.001
Ba (mg L^-1^)	0.00715	0.00401	0.00445	0.014
Pb (mg L^-1^)	0.0029	0.00175	<0.0005	0.00124
Cd (mg L^-1^)	<0.0001	<0.0001	<0.0001	<0.0001
Co (mg L^-1^)	0.000385	0.00250	0.00150	0.00360
Cu (mg L^-1^)	0.72	1.15	0.017	0.047
Cr (mg L^-1^)	<0.0005	<0.0005	<0.0005	0.0011
Hg (mg L^-1^)	<0.0001	<0.0001	<0.0001	<0.0001
Ni (mg L^-1^)	0.275	0.00695	0.00515	0.0079
V (mg L^-1^)	<0.0002	0.0003	<0.0002	0.0018
Zn (mg L^-1^)	0.625	0.45	0.0135	0.0435

TAP, NUT, RW and PW represent tap water, optimal liquid fertilizer, reclaimed effluent water and primarily treated wastewater, respectively.

In addition to the chemical characterization performed on the irrigation samples used in the experiment, operational wastewater-quality data were obtained from the municipal WWTP to provide further context regarding the characteristics of the two wastewater streams. For PW, the available plant data included total N (37.5 mg L^-^¹), NH_4_-N (27 mg L^-^¹), total P (2.9 mg L^-^¹), suspended solids (136 mg L^-^¹), biochemical oxygen demand (BOD; 96 mg L^-^¹), chemical oxygen demand (COD; 256 mg L^-^¹), and a pH of 7.3. For RW, the corresponding available values were total N (13 mg L^-^¹), NH_4_-N (3.8 mg L^-^¹), total P (0.13–0.14 mg L^-^¹), suspended solids (3.5 mg L^-^¹), BOD (3.86 mg L^-^¹), COD (<30 mg L^-^¹), and an average pH of 7.1.

EC, total dissolved solids (TDS), nitrate, and individual phosphate species were not available for both wastewater streams. Dedicated microbiological characterization of the irrigation water and harvested plant material was not performed as part of this experiment.

### 2.3 Growth medium characterization

The substrate selected for this study is a zeolite-based mixture (**Figure 1b**), commercially known as Lechuza BASICPON, composed of peat-free and natural raw minerals, including pumice, lava, and zeolite. Notably, this substrate is unfertilized, providing an ideal foundation for precise fertilization practices. The zeolite was selected due to its unique properties that facilitate accurate nutrient management and simulate conditions typical of hydroponic systems. To establish a controlled baseline for the experiment, the substrate was thoroughly washed. This process involved soaking and rinsing the substrate with distilled water multiple times to remove any residual contaminants or soluble salts that could affect the experimental outcomes. The substrate’s high porosity and cation-exchange capacity (Cataldo et al., 2021; Gül et al., 2005; Harland et al., 1999; Sangeetha and Baskar, 2016) are key properties contributing to its appropriateness for this research. Furthermore, the zeolite component in the substrate enhances its sustainability, as it can be recycled and reused multiple times (Harland et al., 1999). This feature aligns with the circular-based approach of the study and underscores the substrate’s compatibility with the research objectives. The raw zeolite composition analyzed by Eurofins Scientific SE can be found in **Table 2**.

**Figure 1:**
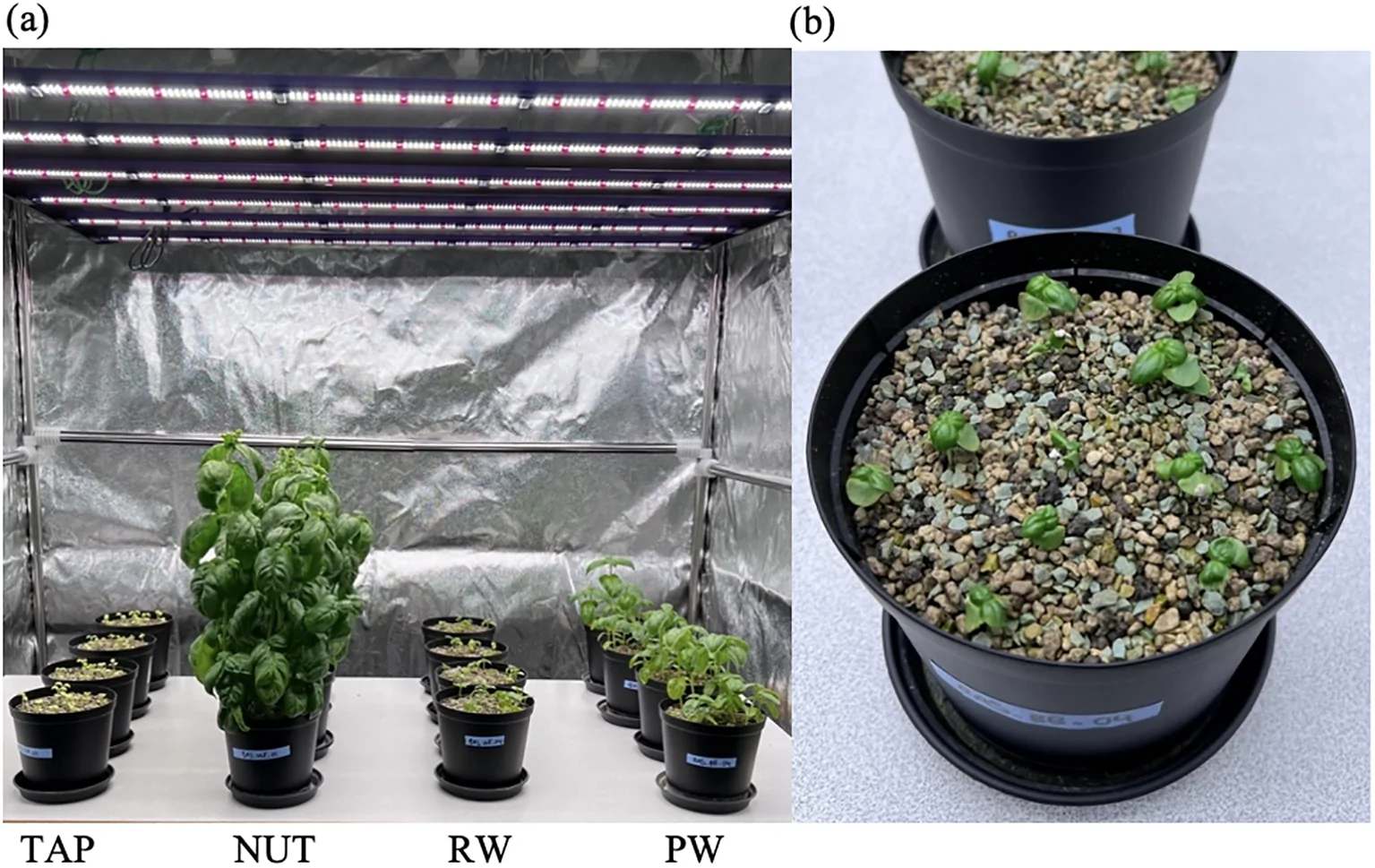
**(a)** Experimental setup showing basil cultivation under different irrigation water sources. TAP, NUT, RW and PW represent tap water, optimal liquid fertilizer, reclaimed effluent water and primarily treated wastewater, respectively. **(b)** Zeolite-based substrate.

**Table 2:** Composition of raw zeolite used in this experiment.

Parameters/Unit	Concentration	Parameters/Unit	Concentration
Arsenic (mg/kg^-1^)	3.10	Nickel (mg/kg)	7.10
Barium (mg/kg)	550	Vanadium (mg/kg)	29
Lead (mg/kg)	6.40	Zinc (mg/kg)	30
Cobalt (mg/kg)	5.20	Phosphorus (mg/kg)	620
Copper (mg/kg)	14	Potassium (mg/kg)	18000
Chromium (mg/kg)	2.90	Dry Matter (%)	80.40

Values for elemental concentrations in zeolite are expressed on a dry-weight basis.

### 2.4 Analysis of plant properties

Before harvesting, plant height was measured from the soil surface to the top of the plant. The number of leaves per plant was counted. After harvesting, plants’ shoots and roots were separated. Fresh biomass of above-ground parts and leaves of each plant were weighted. The samples were then oven-dried at 70 °C for four days until they reached a constant weight. Macro and microelement concentrations in the collected plant samples were subsequently determined on a dry-weight basis by an external laboratory (Eurofins Scientific SE) using inductively coupled plasma mass spectrometry (ICP-MS) and inductively coupled plasma atomic emission spectroscopy (ICP-AES).

### 2.5 Risk assessment

#### 2.5.1 Bioconcentration and translocation factors

The bioconcentration factor (BF), measures elements uptake by plant from substrate through roots and shoots which can give an indication for the accumulation of elements in the different parts of plants (Subramanian et al., 2022). The BC was calculated using the following equation:


$$\text{BF}=\frac{\text{The specific element concentration in shoots and roots}}{\text{The specific element concentration in the substrate}}$$

While the translocation factor (TF) measures the translocation of elements from roots to the edible part of plants the shoots (Usman et al., 2019) and can be calculated by the following equation:


$$\text{TF}=\frac{\text{The specific element concentration in shoots }}{\text{The specific element concentration in roots}}$$

#### 2.5.2 Health risk assessment

Non-carcinogenic health risk assessment of individual metal was calculated as the target hazard quotient (THQ) (Nag and Cummins, 2022) for the consumption of basil shoots for a typical adult in Sweden by assuming an average body weight (BW) of 80 kg and an average life expectancy of 83 years (SCB, 2024). The THQ was calculated based on the following equation:


$$THQ=\frac{EDI}{RFD}$$

Where RFD is oral reference dose for the analyzed metals (mg/kg body weight per day). The RFD for Co, Cu, Cr and Zn was 0.03, 40, 3 and 0.3 (mg/kg/day) respectively, based on the collected data from the United States Environmental Protection Agency (USEPA, 2021).

While EDI can be calculated using the following equation:


$$EDI=\frac{C_{p}\;.\;ADC.E_{F\;}.ED}{BW.AT}$$

Where:

EDI is the estimated daily intake of each individual metal (mg/kg body weight per day); Cp concentration of individual metal in shoots (mg/g); ADC is the average daily consumption of basil (g/day), according to the European Food Safety Authority (EFSA) the average daily consumption of basil is around 3 g/day; EF is the exposure frequency (365 day/year); ED represents the exposure duration of adult in Sweden (83 years); AT is the exposure average time (day) calculated through the multiplication of consumption days per year by the exposure duration.

The THQ numerical value assesses the potential non-carcinogenic health risks, if the values are less than 1 then no evident adverse heath effect can be expected while values higher than 1 will be related to clear non-carcinogenic health effects (Wieczorek et al., 2023). In addition, if the summation of all THQ of all elements in the same water type exceeding 1 then obvious adverse health effect can be expected (Wieczorek et al., 2023).

### 2.6 Statistical analysis

Data are presented as mean ± standard deviation. To discern significant differences among treatment groups, one-way analysis of variance (ANOVA) was performed. When the ANOVA indicated a significant treatment effect, Duncan’s multiple range test was applied for *post-hoc* comparisons. Variables available for only two treatment groups were compared using an independent-samples Student’s t-test. Before conducting parametric analyses, normality and homogeneity of variance were assessed using the Shapiro–Wilk and Levene’s tests, respectively. When homogeneity of variance was violated, Welch’s ANOVA followed by Games–Howell *post-hoc* comparisons was applied. Where normality was not initially satisfied, data were log-transformed and assumptions were reassessed before parametric analysis. The significance level was set at 0.05. Excel 2024 and SPSS 28.0.1.0 (142) software packages were used for statistical analyses, while Sigmaplot 14.0 was employed to generate figures.

## 3 Results

### 3.1 Basil production under different irrigation water sources

Growth chlorosis was observed with plants irrigated with tap water (TAP) and reclaimed effluent water (RW) throughout the entire growth stage (**Figure 1A**). In contrast, better plant growth and development were visually observed in the primarily treated wastewater (PW) and optimal liquid fertilizer (NUT) treatments compared to TAP and NUT treatments. With the optimal nutrient solution, the highest fresh shoot weight of basil was observed in the NUT treatment (**Figure 2C**). A similar phenomenon was observed for plant height, the number of leaves per plant and the fresh weight of one leave, with the highest values consistently observed in the NUT treatment (**Figures 2A, B, D**).

**Figure 2:**
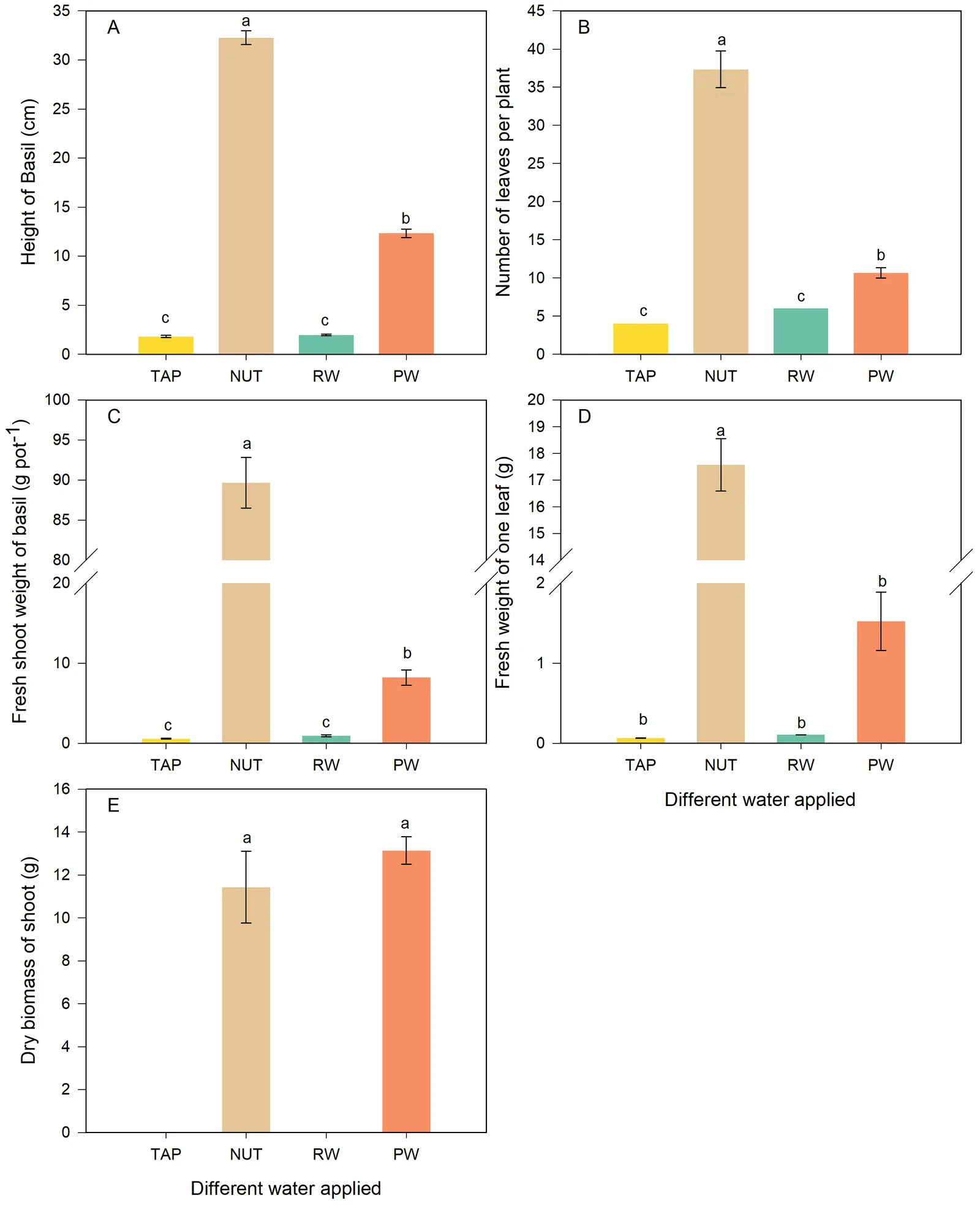
Biomass production under different irrigation water sources. **(A)** Height of basil, **(B)** Number of leaves per plant, **(C)** Fresh shoot weight of basil, **(D)** Fresh weight of one leaf, **(E)** Dry biomass of shoot. TAP, NUT, RW and PW represent tap water, optimal liquid fertilizer, reclaimed effluent water and primarily treated wastewater, respectively. Different lowercase letters indicate significant differences among treatments at P < 0.05. Data are presented as mean ± standard deviation (n=3).

When utilizing the treated wastewater, the PW treatment evidently increased the fresh shoot biomass from 0.5754 to 8.2033g, corresponding to a 1326% increase compared with the TAP treatment (P < 0.05; **Figure 2C**). Additionally, plant height and leaf number were notably enhanced by 585% and 167%, respectively, with the PW treatment compared to TAP (**Figures 2A, B**). Conversely, the use of treated wastewater RW resulted in similar fresh biomass production, plant height and leaf numbers to TAP.

Due to the limited biomass production from TAP and RW treatments, only dried biomass from NUT and PW treatments were presented (**Figure 2E**). Interestingly, no significant difference was observed in dry biomass between the NUT and PW treatments.

### 3.2 Effect of wastewater irrigation on macronutrients in basil

Macronutrient (N, P, K) concentrations in basil shoots and roots varied depending on the type of irrigation water used (**Figure 3**). Due to a limited sample size, N concentrations were measured only for basil shoots with the NUT and PW treatments. No significant difference was observed between these treatments (**Figure 3A**).

**Figure 3:**
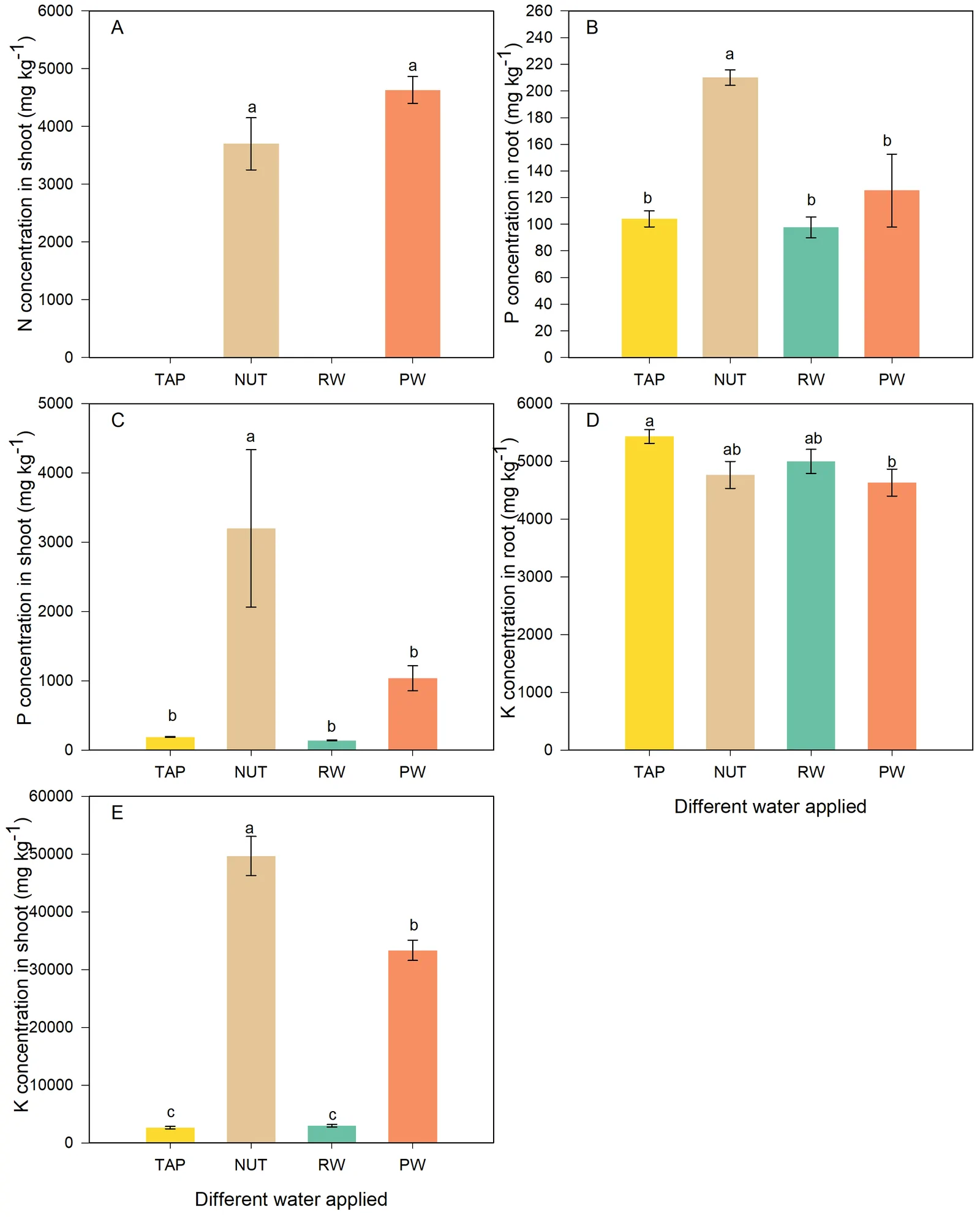
Concentrations of macronutrients in shoots and roots of basil under different irrigation water sources, expressed on a dry-weight basis. **(A)** N concentration in shoot of basil, **(B)** P concentration in root of basil, **(C)** P concentration in shoot of basil, **(D)** K concentration in root of basil, **(E)** K concentration in shoot of basil. TAP, NUT, RW and PW represent tap water, optimal liquid fertilizer, reclaimed effluent water and primarily treated wastewater, respectively. Different lowercase letters indicate significant differences among treatments at P < 0.05. Data are presented as mean ± standard deviation (n=3).

Basil grown with the NUT treatment exhibited the highest P concentrations in both shoots and roots (**Figures 3B, C**). In contrast, basil irrigated with both treated wastewater RW and PW showed similar P concentrations in shoots and roots to those in the TAP treatment.

Regarding K, the highest concertation in shoots was observed with the NUT treatment, followed by the PW treatment. With the PW wastewater irrigation, K concentrations in basil shoot were significantly increased by 1166% and 1036%, respectively, compared to that with TAP and RW. Interestingly, irrigation with RW wastewater exhibited similar K concentrations in basil roots compared to those in NUT and TAP treatments. However, the PW treatment resulted in a significant 17% decrease in root potassium (K) concentration compared to TAP. Nevertheless, there was no evident difference in root K concentration among PW, NUT, and RW treatments.

### 3.3 Effect of wastewater irrigation on micronutrients in basil

The concentrations of micronutrients (Cu, Zn, Mn) in basil shoots and roots affected by different irrigation waters are presented in **Figures 4**, **5**, respectively. With the application of treated wastewater, Mn concentrations in basil shoots with PW and RW treatments were similar to those in NUT and TAP, respectively, with the highest Mn concentrations observed with PW and NUT. For instance, Mn levels in basil irrigated with PW wastewater evidently increased by 308% and 474%, respectively, compared to that with TAP and RW. However, basil grown with NUT exhibited similar Mn concentrations in the roots as TAP, while basil irrigated with PW showed 229% and 170% higher Mn concentrations in roots than TAP and NUT, respectively.

**Figure 4:**
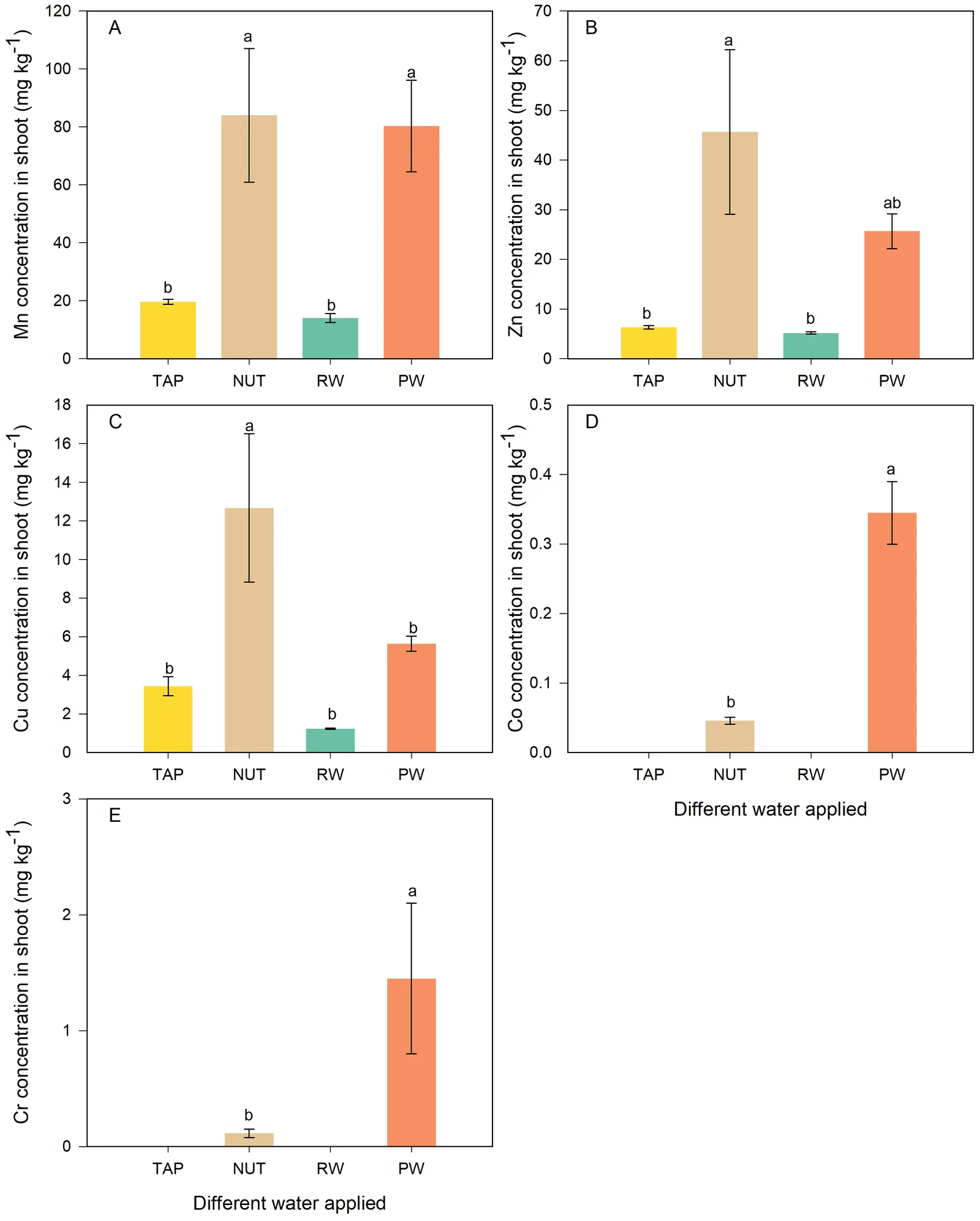
Concentrations of micronutrients in basil shoots under different irrigation water sources, expressed on a dry-weight basis. **(A)** Mn concentration in shoot of basil, **(B)** Zn concentration in shoot of basil, **(C)** Cu concentration in shoot of basil, **(D)** Co concentration in shoot of basil, **(E)** Cr concentration in shoot of basil. TAP, NUT, RW and PW represent tap water, optimal liquid fertilizer, reclaimed effluent water and primarily treated wastewater, respectively. Different lowercase letters indicate significant differences among treatments at P < 0.05. Data are presented as mean ± standard deviation (n=3).

**Figure 5:**
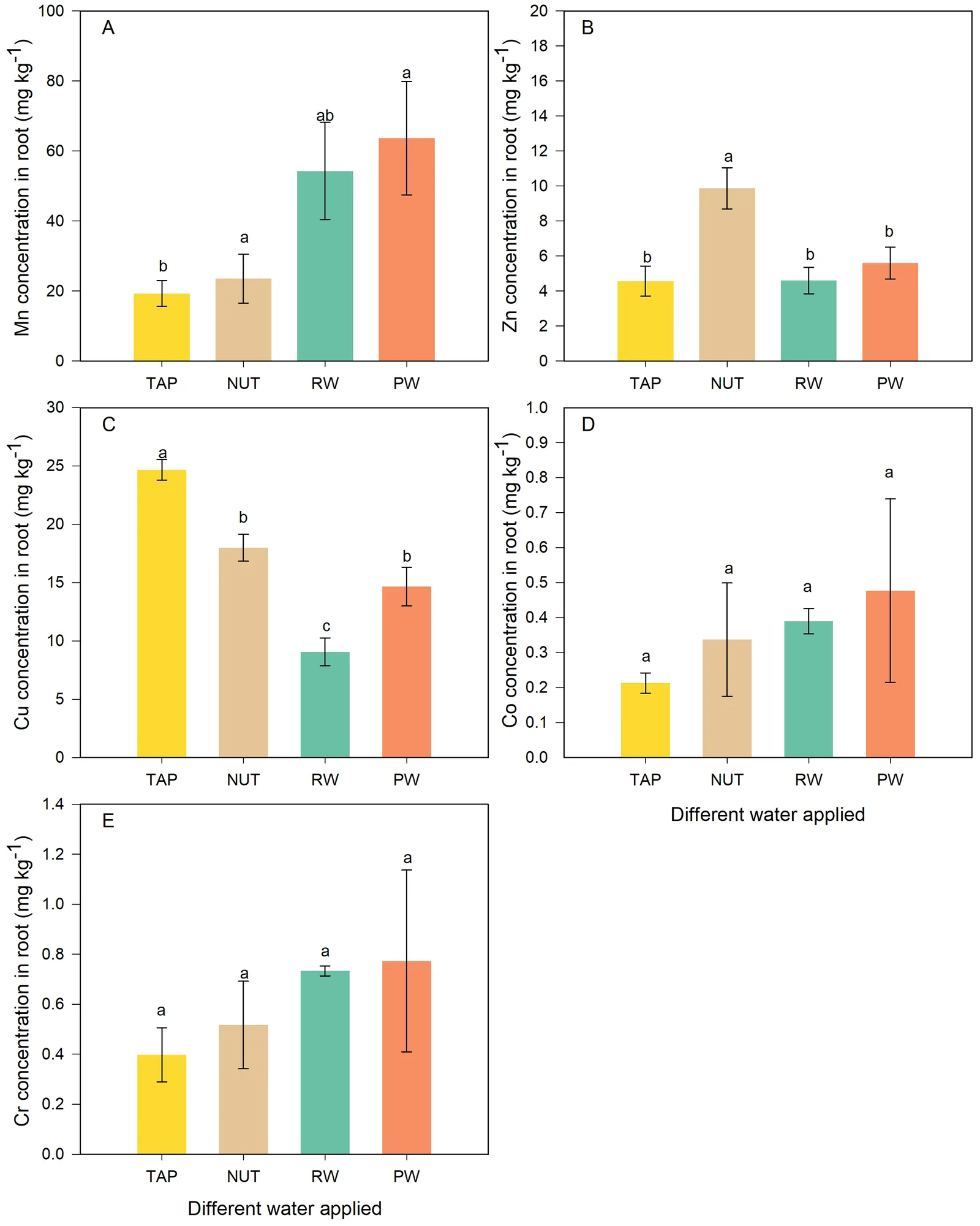
Concentrations of micronutrients in basil roots under different irrigation water sources, expressed on a dry-weight basis. **(A)** Mn concentration in root of basil, **(B)** Zn concentration in root of basil, **(C) ** Cu concentration in root of basil, **(D)** Co concentration in root of basil, **(E)** Cr concentration in root of basil. TAP, NUT, RW and PW represent tap water, optimal liquid fertilizer, reclaimed effluent water and primarily treated wastewater, respectively. Different lowercase letters indicate significant differences among treatments at P < 0.05. Data are presented as mean ± standard deviation (n=3).

Basil irrigated with PW wastewater showed similar Zn concentration in the shoots to those with NUT, whereas basil irrigated with RW wastewater exhibited similar Zn concentrations to those irrigated with TAP. Moreover, there was no significant difference among shoot Zn concentration with TAP, PW and RW treatments. When considering the roots, Zn concentrations in basil irrigated with PW and RW wastewater were similar to those irrigated with TAP water. In summary, the micronutrient Zn levels in basil irrigated with PW and RW wastewater were comparable to those in basil irrigated with TAP water.

Similar to Zn, there was no significant difference among shoot Cu concentration with TAP, PW and RW treatments. However, in the roots, the highest Cu concentration was observed in the TAP treatment. Cu concentrations in roots irrigated with NUT, RW, and PW were significantly decreased by 37%, 172%, and 68%, respectively, compared to TAP. Nevertheless, PW irrigation yielded similar Cu concentrations in roots as NUT.

There is no significant difference in Co and Cr concentrations in basil roots among the four irrigation water sources (**Figures 5D, E**). However, PW resulted in higher Co and Cr concentrations in basil shoots compared to NUT (**Figures 4D, E**).

Overall, irrigation with PW wastewater resulted in higher levels of micronutrients in basil shoots compared to TAP, while RW exhibited similar micronutrient levels to TAP. However, treatments, including PW, TAP, and RW, require supplementation of certain elements such as Cu and Zn to achieve micronutrient levels comparable to those in the NUT treatment.

### 3.4 Health risk assessment

In this section, the focus was on assessing the potential health risks associated with the four elements Co, Cu, Cr, and Zn in the edible parts of basil, the shoots, as the accumulation of these elements may directly affect human health. Results of the TF and BF of the shoots are shown in **Table 3**. TF results indicated that the elements accumulated in the shoots were Co, Cu, Cr and Zn. The major concentration was for the Zn in the shoots with (4.646 ± 0.450) in NUT water. Different accumulation behavior was observed for each one of the studied elements depending on the initial concentration in the water sample as well as the ability of basil to transfer the metal under consideration to the shoots. For instance, concentrations of Co in TAP water samples were not detectable compared to that in NUT, RW or PW while Cu was available in all samples of the different waters. Results of the BC showed the same behavior of TF with Zn dominating with (1.268E + 00 ± 8.338E-01) in NUT water. High value of BF means that the Basil has the ability to stabilize and accumulate the element in the shoots system.

**Table 3:** Average values of Translocation factor (TF) and Bioconcentration factor (BF) (± standard deviation) of the basil with focus on the four studied metals effecting risk assessment.

Treatments	Translocation factor			
	Cobalt (Co)	Copper (Cu)	Chromium (Cr)	Zinc (Zn)
TAP	ND	0.139 ± 0.032	0.109 ± 0.189	1.514 ± 0.601
NUT	0.004 ± 0.006	0.734 ± 0.428	0.246 ± 0.426	4.536 ± 2.289
RW	0.005 ± 0.002	0.142 ± 0.041	0.102 ± 0.103	1.198 ± 0.363
PW	0.016 ± 0,015	0.388 ± 0.030	2.293 ± 2.392	4.646 ± 0.450
Bioconcentration factor				
TAP	ND	1.974E-01 ± 3.965E-02	2.667E-02 ± 3.771E-02	1.710E-01 ± 9.451E-03
NUT	8.458E-03 ± 1.465E-02	4.824E-01 ± 2.451E-01	6.263E-02 ± 1.085E-01	1.268E+00 ± 8.338E-01
RW	7.877E-03 ± 1.484E-03	8.603E-02 ± 5.499E-04	2.283E-02 ± 2.210E-02	1.419E-01 ± 1.195E-02
PW	3.667E-02 ± 3.215E-02	3.403E-01 ± 5.838E-02	2.862E-01 ± 3.230E-01	7.083E-01 ± 1.275E-01

ND means not detected, the analyte concentration was below the method detection limit. TAP, NUT, RW and PW represent tap water, optimal liquid fertilizer, reclaimed effluent water and primarily treated wastewater, respectively. Data are presented as mean ± standard deviation (n=3).

**Table 4** presents the EDI and THQ results of the four metals (Co, Cu, Cr and Zn) in Basil shoots. The non-carcinogenic health risks associated with the accumulation of these elements in the shoots (based on the EDI, THQ and ∑THQ) are considered irrelevant as the EDI, THQ and ∑THQ values were all less than 1 for all the metals. The THQ values of the Co ranged between zero in TAP water to (2.875E-04 ± 2.553E-04) in PW water and with the order PW> NUT> RW> TAP. For the Cu the results ranged between (1.156E-06± 5.413E-08) in RW water to (1.188E-05± 6.242E-06) in NUT water and the order NUT> PW> TAP> RW. Different behavior was observed for the THQ values of the Cr with the order PW> NUT> RW> TAP ranging between (8.333E-07± 1.443E-06) in TAP water to (1.208E-05± 1.325E-05) in PW water. While the order regarding the Zn accumulated in the Basils shoots was NUT> PW> TAP> RW ranging between (6.500E-04± 5.000E-05) in RW to (5.708E-03± 3.585E-03) in NUT.

**Table 4:** Results of EDI and THQ for the four studied metals in the Basils shoots.

Treatments	EDI (mg/kg.day)				THQ				∑ THQ
	Co	Cu	Cr	Zn	Co	Cu	Cr	Zn	
TAP	ND	1.288E-04± 3.189E-05	2.500E-06± 4.330E-06	2.375E-04± 2.440E-05	ND	3.219E-06± 7.973E-07	8.333E-07± 1.443E-06	7.917E-04± 8.133E-05	7.957E-04
NUT	2.125E-06± 3.681E-06	4.750E-04± 2.497E-04	7.750E-06± 1.342E-05	1.713E-03± 1.075E-03	7.083E-05± 1.227E-04	1.188E-05± 6.242E-06	2.583E-06± 4.474E-06	5.708E-03± 3.585E-03	5.794E-03
RW	1.725E-06± 3.070E-07	4.625E-05± 2.165E-06	2.850E-06± 2.813E-06	1.950E-04± 1.500E-05	5.750E-05± 1.023E-05	1.156E-06± 5.413E-08	9.500E-07± 9.377E-07	6.500E-04± 5.000E-05	7.096E-04
PW	8.625E-06± 7.658E-06	2.113E-04± 2.553E-05	3.625E-05± 3.975E-05	9.625E-04± 2.260E-04	2.875E-04± 2.553E-04	5.281E-06± 6.381E-07	1.208E-05± 1.325E-05	3.208E-03± 7.535E-04	3.513E-03

ND means not detected, the analyte concentration was below the method detection limit. TAP, NUT, RW and PW represent tap water, optimal liquid fertilizer, reclaimed effluent water and primarily treated wastewater, respectively. THQ means target hazard quotient and EDI is the estimated daily intake of each individual metal. Data are presented as mean ± standard deviation (n=3).

## 4 Discussion

### 4.1 Biomass production under different irrigation water conditions

Wastewater irrigation has been proposed as a strategy to support plant growth while addressing water scarcity. In this study, basil irrigated with PW wastewater showed a significant improvement in growth parameters and visual appearances compared to RW and TAP (**Figures 1**, **2**). Although shoot biomass under PW irrigation was 1326% higher than that under TAP irrigation, this large relative increase was partly driven by the extremely low biomass (0.5754 g) observed in the TAP treatment and should therefore be interpreted cautiously. Nevertheless, the greater biomass production under PW irrigation suggests that partially treated wastewater has the potential to be reused to support positive plant growth compared to the other two water source. Similar results have been observed by Manios et al. (2006) for using primary-treated wastewater, where higher nitrogen content of this stream of wastewater led to significant yield increases in crops like carrots, sugar beet, and potatoes, compared to secondary treated wastewater and tap water. Besides, the similarity in dry matter content between the NUT and PW treatments underscores that partially treated wastewater may have supported biomass accumulation comparable to the conventional nutrient solution (**Figure 2**). The increase in biomass production by PW can be attributed to the relatively higher nutrients compared to TAP (**Table 1**). This can be further proved by the higher nutrient content in basil shoots (**Figure 4**). This suggests that PW water has potential as a supplementary irrigation source for enhancing basil growth and production. However, the NUT treatment consistently produced the greatest growth across several parameters, including fresh and dry weights of shoots, individual leaf weight, leaf number, and shoot height (**Figure 2**), and thus, PW should not be considered a complete substitute for a balanced nutrient solution (NUT), and additional nutrient supplementation required to achieve optimal plant growth.

When irrigated with RW, basil exhibited similar growth chlorosis and the lowest fresh shoot weight, leaf numbers, and shoot height as observed with TAP irrigation. These findings are consistent with previous studies suggesting that treated wastewater alone may not provide sufficient nutrients for optimal plant growth. For example, Adrover et al. (2013) found that barley grown in hydroponic conditions with treated wastewater showed a significant reduction in biomass compared to those grown with a nutrient solution, mainly due to lower concentrations of essential nutrients such as nitrogen and iron in the wastewater. Additionally, Cifuentes-Torres et al. (2021) highlighted the challenges of using RW in hydroponic systems without nutrient supplementation. While RW can provide some essential nutrients, studies have shown that it often fails to supply the full spectrum required for optimal plant growth. For instance, Morillo et al. (2009) observed that cucumbers irrigated with untreated RW exhibited chlorosis, necrosis, and limited growth, likely due to inadequate potassium levels or other trace elements. Similarly, Boyden and Rababah (1996) found that lettuces grown with wastewater were smaller and did not reach maturity, underscoring the need for additional nutrient inputs. This suggests the need for spiking the wastewater with additional nutrients to meet the plants’ needs (Adrover et al., 2013; Cifuentes-Torres et al., 2021). This is further supported by our results, where the NUT treatment consistently led to the highest basil production across multiple growth parameters, including fresh and dry weights of shoots, individual leaf weight, leaf number, and shoot height (**Figure 2**). The superior growth observed in the NUT treatment may be explained by its more balanced and readily available nutrient composition (**Table 1**), which promote vigorous plant growth (Langenfeld et al., 2022; Rouphael et al., 2012, 2018).

Recent studies similarly show that crop performance depends strongly on wastewater treatment stage and nutrient composition. Santos et al. (2024) compared primary- and secondary-treated wastewater with a conventional nutrient solution for hydroponic lettuce cultivation and reported acceptable growth with both wastewater streams, although fresh biomass remained lower than in the nutrient-solution treatment. Growth with secondary-treated wastewater was mainly limited by nutrient deficiency, whereas primary-treated wastewater performance was additionally influenced by pH, suspended solids, and nitrite concentration.

### 4.2 Macro and micronutrient concentrations in basil irrigated with different water sources

Wastewater reuse in irrigation provides essential macro- and micronutrients for plant growth, while also affecting nutrient dynamics within the plant. However, the nutrient composition of wastewater can vary, potentially requiring additional supplementation to ensure balanced plant nutrition.

Cu concentrations were highest in the shoots of the NUT treatment, while the highest root concentrations were observed in the TAP treatment. This may be associated with translocation mechanisms, where excess Cu is often sequestered in the roots to avoid toxicity in the shoots. Plants often regulate Cu uptake to prevent toxicity, and when exposed to high concentrations of Cu in the environment, they tend to accumulate it in the roots (Ghazaryan et al., 2019; Ogunkunle et al., 2019). Additionally, the pH of the growing medium can influence Cu mobility. The lower pH levels in NUT (pH = 6) compared to other treatments, resulting a higher Cu solubility and bioaccessibility, may facilitate greater translocation into shoots (Alva et al., 2000; Shabbir et al., 2020). Co and Cr were present originally in the raw zeolite (5.2 mg/kg for Co and 2.9 mg/kg for Cr). In the irrigation sources, Co was present in all treatments, with the highest levels in the PW treatment, while Cr was detected only in PW. As a result, both Co and Cr were found across all roots, likely due to the original levels in the substrate. However, only the wastewater treatments (PW and RW) contained detectable levels of Co and Cr in the shoots, with significantly higher concentrations in the PW treatment. Accumulation of Co and Cr in the roots of NUT and TAP treatments rather than shoots could be associated with a plant defense mechanism, limiting shoot exposure to toxicity. Zayed et al. (1998) conducted hydroponic studies on various vegetable species and found that Cr’s transformation and strong binding in roots, restricting its upward translocation. Using X-ray absorption spectroscopy, they showed that Cr (VI) is reduced to Cr (III) in the roots, where it binds to cell walls, resulting in root concentrations up to 100 times higher than in shoots. Similarly, Lotfy and Mostafa (2014) found that roots accumulated more Co and Cr than shoots in several plant species, mainly due to the higher bioavailability of them in the substrate and their limited translocation to shoots. Other metals such as Pb, As, Ni and V were found only in the roots, with no significant differences in their concentrations among different treatments. This may indicates that these metals were taken up by the plants but not translocated to the shoots, potentially as a mechanism to avoid toxicity in the aerial parts of the plant. The study by Souri et al. (2019) supports this observation, indicating that root concentrations of various heavy metals, particularly Cd, As, Ni, Co, Cu, Mn, and Zn, were significantly higher than their shoot concentrations in both garden cress and sweet basil. This may be attributed to the lower mobility of these heavy metal cations in plants, leading to their accumulation in the roots rather than translocation to the shoots. Also, since roots are the initial point of contact with heavy metal ions, the duration of exposure could influence the final concentration (Souri et al., 2019).

### 4.3 Substrate analysis

Post-experiment substrate analysis showed higher accumulation of various nutrients and elements including P, K, Pb, Ba, Cu, Ni, and Zn compared to raw zeolite across treatments, especially in NUT treatment. There was a notable accumulation of K in the substrate compared to the raw substrate. One reason for this could be the high cation exchange capacity of zeolite (Cataldo et al., 2021; Gül et al., 2005; Harland et al., 1999; Sangeetha and Baskar, 2016), so its exchangeable sites can act as a reservoir for potassium, adsorbing excess soluble K and making it available to plants when soil K levels drop (Li et al., 2022) Additionally, the high cation exchange capacity of zeolite means it can also hold other essential nutrients, slowly releasing them into the rhizosphere as needed. This ability could enhance the overall nutrient uptake by plants. The findings from Gül et al. (2005) suggests the same, indicating that the increased nutrient uptake observed is consistent with the element retention properties of zeolite, which acts as a slow-release reservoir, reducing nutrient leaching and providing a sustained nutrient supply to plants over time. This dynamic adsorption and release process mitigates the risk of K being fixed by minerals or organic matter (Eslami et al., 2020).

### 4.4 Environmental, health, and sustainability implications

One of the main concerns regarding wastewater reuse for irrigation is the accumulation of contaminants in edible plant tissues and their potential transfer to the food chain. In the current study, the low EDI, THQ, and cumulative THQ values indicate a low estimated non-carcinogenic risk from the four selected metals under the tested exposure assumptions. Similarly, Lee et al. (2021) reported health risk index and THQ values below 1 for heavy metals in lettuce cultivated hydroponically with anaerobically treated wastewater, indicating a low estimated non-carcinogenic risk under their experimental conditions. However, the present findings apply only to the selected metals analyzed and should not be interpreted as confirmation of comprehensive food safety.

From an agronomic perspective, our findings demonstrate the potential of using treated wastewater, particularly PW, as a supplementary source of certain nutrients and irrigation water rather than as a direct replacement for conventional fertilizers. The comparable growth performance and nutrient uptake observed in the PW treatment suggest that with appropriate management, wastewater can provide essential nutrients while reducing reliance on synthetic fertilizers. Nevertheless, nutrient balancing and supplementation would be required to achieve consistently optimal crop production.

Despite its agronomic potential, PW cannot be recommended in its present form for the direct commercial irrigation of raw-consumed basil. Regulation (EU) 2020/741 requires reclaimed water used for above-ground food crops consumed raw to undergo at least secondary treatment and disinfection, including when the irrigation method avoids direct contact with the edible parts of the crop. Because PW had undergone only mechanical treatment, it did not meet these requirements. The root-zone application used in the present pot experiment prevented direct contact between the irrigation water and basil shoots and therefore reduced one potential exposure pathway. However, this configuration could not eliminate risks associated with pathogens, internal contaminant uptake, handling, cross-contamination, or contaminants of emerging concern. It should therefore be regarded as one possible component of a multi-barrier risk-management approach rather than as a substitute for adequate wastewater treatment (European Parliament and Council of the European Union, 2020).

Municipal wastewater can contain a broader spectrum of contaminants and pathogens that were not addressed here. These include emerging micropollutants, organic contaminants, pathogenic microorganisms and antibiotic resistance genes (ARGs) (Narwal et al., 2023). Emerging micropollutants such as pharmaceuticals, personal care products, hormones and microplastics are increasingly detected in municipal wastewater. These substances can persist through conventional treatment processes and may accumulate in edible plant tissues, posing potential risks to human health and the environment. Organic contaminants such as pesticides, industrial chemicals, and disinfection by-products may also be present in trace amounts. Their uptake and effects on crops and consumers remain largely uncharacterized in the context of wastewater reuse. Pathogenic Microorganisms such as bacteria (e.g., E. coli, Salmonella), viruses, and protozoa can survive wastewater treatment, especially in partially treated streams. Recent evidence nevertheless indicates that controlled environment cultivation may reduce certain exposure pathways. Santos et al. (2024) cultivated lettuce hydroponically using primary- and secondary-treated municipal wastewater and assessed total coliforms and E. coli. Although coliforms were mainly detected in the roots, E. coli was not detected on the edible leaves, total coliform concentrations in the leaves remained within the limits applied in that study. Although indoor farming and controlled cultivation without direct contact between wastewater and edible tissues may reduce some risks, the potential for pathogen transfer to crops and subsequent human exposure cannot be excluded. Finally, wastewater is a known reservoir for ARGs, which can be transferred to soil and plant microbiomes, potentially contributing to the spread of antimicrobial resistance.

From a resource-recovery perspective, the results indicate that wastewater streams collected before biological nutrient removal warrant further investigation as feedstocks for targeted reclamation processes. Rather than applying PW directly, future treatment steps could be designed to retain recoverable nitrogen and other nutrients while selectively reducing pathogenic microorganisms and relevant chemical hazards. Such systems would require nutrient balancing, validated disinfection, microbiological monitoring, analysis of contaminants of emerging concern, and verification of compliance with applicable water-reuse and food-safety requirements before practical application. The present findings provide preliminary agronomic and selected-metal data that may support the development and future risk-based evaluation of nutrient-preserving treatment strategies; however, they are not sufficient on their own to establish safety or support changes to existing regulations.

Several considerations should be taken into account when interpreting the findings. The experiment included three independent replicate pots per treatment. Although this replication was sufficient to detect the large treatment effects observed for several growth parameters, it may have limited the statistical power to identify smaller differences in nutrient and metal concentrations. In addition, only one basil cultivar and one cultivation cycle were evaluated. The controlled indoor pot system was intentionally used to reduce environmental variability and isolate the effects of irrigation-water composition; however, it does not fully reproduce the scale, nutrient recirculation, microbial dynamics, or management conditions of commercial hydroponic production. Further studies involving additional replicates, cultivars, cultivation cycles, and pilot-scale systems are therefore required to evaluate the wider applicability of the findings.

Future studies should address the mentioned limitations and also include a broader spectrum of contaminants and diverse crops providing a full risk profile of wastewater reuse. Research should focus on optimizing wastewater treatment processes to selectively remove hazardous contaminants while retaining beneficial nutrients. Treatment approaches such as ozonation, disinfection, filtration, or other targeted processes should be evaluated for their ability to reduce microbial and chemical hazards without unnecessarily removing recoverable nutrients. Tailored nutrient supplementation (“spiking”) may be necessary to achieve optimal crop performance. The long-term effects of wastewater irrigation on substrate quality, nutrient accumulation, microbial communities, and contaminant transfer should also be assessed. Furthermore, the dual role of crops in nutrient recovery and phytoremediation could be assessed as it might enhance the value proposition of wastewater reuse, provided that the intended use and safe management of harvested biomass are clearly established.

## 5 Conclusions

This study highlights the potential of using treated wastewater in agriculture while addressing its challenges. Copper and zinc concentrations in basil were elevated in positive controls and PW treatments but remained below contamination limits, emphasizing the need for monitoring and management of synthetic fertilizers in indoor food productions settings. Cobalt and chromium concentrations in wastewater-treated plants were within acceptable limits, and heavy metals like mercury, nickel, arsenic, and lead were below detection limits in edible parts of plant. These findings indicate a low non-carcinogenic risk for the selected metals under the tested conditions but should not be interpreted as confirmation of overall food safety.

In this study, PW showed potential as a supplementary source of irrigation water and nitrogen for basil production with the outperformed several growth parameters compared to RW and TAP. However, the NUT treatment generally resulted in greater basil growth, indicating that treated wastewater cannot fully replace a balanced nutrient solution and additional nutrient supplementation may therefore be required to achieve optimal production.

Despite its agronomic potential, PW cannot be recommended in its present form for the commercial irrigation of raw-consumed crops because it does not meet current EU treatment requirements and was not evaluated for microbiological hazards or contaminants of emerging concern. Although root-zone irrigation prevented direct contact between the wastewater and edible shoots, additional treatment, validated disinfection, nutrient balancing, monitoring, and comprehensive safety assessment would be required before practical application. Future research should evaluate nutrient-preserving treatment strategies and validate the findings using additional cultivars, longer cultivation periods, broader contaminant analyses, and pilot-scale controlled-environment systems.

## Data Availability

The raw data supporting the conclusions of this article will be made available by the authors, without undue reservation.

## Supplementary material

The Supplementary Material for this article can be found online https://www.frontiersin.org/articles/10.3389/fpls.2026.1919603/full#supplementary-material
