# Supplementary material for: Nutrient recovery from treated municipal wastewater for basil production in indoor farming
Source: Front Plant Sci. 2026 Sep 17;17:1919603. doi: 10.3389/fpls.2026.1919603 (PMC13627342; doi:10.3389/fpls.2026.1919603)
Supplement: Supplementary file 1 [file SupplementaryFile1.docx]

**Supplementary materials**

**Nutrient Recovery from Treated Municipal Wastewater for Basil production in Indoor Farming**

Reyhaneh Gorji^1*^, Xiaolin Wang^1*^, Yahya Jani^1^, Sebastian Schwede^1^, Monica Odlare^1^

^1^ Future Energy Center, Department of Engineering Sciences, Mälardalen University, Västerås, Sweden.

^*^[Reyhaneh.Gorji@mdu.se](mailto:Reyhaneh.Gorji@mdu.se); ^*^x[iaolin.wang.93@gmail.com](mailto:iaolin.wang.93@gmail.com);

Fig. S1. Fresh weight of one leaf production under different irrigation water sources

Fig. S2. Vanadium concentration in basil roots under different irrigation water sources

Fig. S3. Lead concentration in basil roots under different irrigation water sources

Fig. S4. Arsenic concentration in basil roots under different irrigation water sources

## Effect of wastewater irrigation on substrate quality

The analysis of the raw substrate is presented in Section 2.2. Following the experiment, the substrate was analyzed again to determine the accumulation of heavy metals and verify whether the hypothesis that some metals would accumulate in the substrate, potentially as a defense mechanism by the roots to avoid toxicity, holds true. The results indicated that there was no significant difference in K concentrations among treatments; however, there was a notable accumulation of K in the substrate compared to the raw substrate. Regarding heavy metals, including As, Ba, Pb, Co, Cr, Ni, V, and Zn, there were no significant differences in their concentrations among the treatments in the substrate. P concentrations were highest in the NUT treatment, lowest in the RW treatment, and intermediate in the PW and TAP treatments. Cu concentrations were highest in the NUT treatment, whereas no significant differences were observed among the other treatments. The dry matter content was highest in the NUT treatment, while it remained similar across the other treatments.

|  | **TAP** | **NUT** | **RW** | **PW** |
| --- | --- | --- | --- | --- |
| **Dry Matter (%)** | 74,00 ^b^ | 89,30 ^a^ | 74,63 ^b^ | 75,50 ^b^ |
| **Phosphorus (mg/kg Ts)** | 646,66 ^ab^ | 793,33 ^a^ | 590,00 ^b^ | 703,33 ^ab^ |
| **Potassium (mg/kg Ts)** | 19666,66 ^a^ | 20333,33 ^a^ | 19666,67 ^a^ | 19000 ^a^ |
| **Arsenic (mg/kg Ts)** | 3,56 ^a^ | 3,30 ^a^ | 3,40 ^a^ | 3,40 ^a^ |
| **Barium (mg/kg Ts)** | 560,00 ^a^ | 626,66 ^a^ | 536,66 ^a^ | 570,00 ^a^ |
| **Lead (mg/kg Ts)** | 7,60 ^a^ | 6,86 ^a^ | 7,80 ^a^ | 7,93 ^a^ |
| **Cobalt (mg/kg Ts)** | 5,63 ^a^ | 6,56 ^a^ | 5,90 ^a^ | 6,43 ^a^ |
| **Copper (mg/kg Ts)** | 17,66 ^b^ | 26,00 ^a^ | 14,33 ^b^ | 16,66 ^b^ |
| **Chromium (mg/kg Ts)** | 2,96 ^a^ | 3,60 ^a^ | 3,26 ^a^ | 3,30 ^a^ |
| **Nickel (mg/kg Ts)** | 7,76 ^a^ | 8,00 ^a^ | 7,36 ^a^ | 7,70 ^a^ |
| **Vanadium (mg/kg Ts)** | 26,33 ^a^ | 31,66 ^a^ | 26,00 ^a^ | 28,00 ^a^ |
| **Zinc (mg/kg Ts)** | 37,00 ^a^ | 36,33 ^a^ | 36,66 ^a^ | 36,00 ^a^ |

TAP, NUT, RW and PW represent tap water, optimal liquid fertilizer, reclaimed effluent water

and primarily treated wastewater, respectively.
